# Pathogenesis of Multiple Organ Failure: The Impact of Systemic Damage to Plasma Membranes

**DOI:** 10.3389/fmed.2022.806462

**Published:** 2022-03-15

**Authors:** Andrey V. Kozlov, Johannes Grillari

**Affiliations:** ^1^Ludwig Boltzmann Institute for Traumatology, The Research Center in Cooperation With AUVA, LBG, Vienna, Austria; ^2^Austrian Cluster for Tissue Regeneration, Medical University of Vienna, Vienna, Austria; ^3^Laboratory of Navigational Redox Lipidomics and Department of Human Pathology, IM Sechenov Moscow State Medical University, Vienna, Austria; ^4^Institute of Molecular Biotechnology, University of Natural Resources and Life Sciences, Vienna, Austria

**Keywords:** multiple organ failure, plasma membrane, reactive oxygen species, phospholipase A2, pore forming protein, systemic inflammation and sepsis, hypoxia

## Abstract

Multiple organ failure (MOF) is the major cause of morbidity and mortality in intensive care patients, but the mechanisms causing this severe syndrome are still poorly understood. Inflammatory response, tissue hypoxia, immune and cellular metabolic dysregulations, and endothelial and microvascular dysfunction are the main features of MOF, but the exact mechanisms leading to MOF are still unclear. Recent progress in the membrane research suggests that cellular plasma membranes play an important role in key functions of diverse organs. Exploration of mechanisms contributing to plasma membrane damage and repair suggest that these processes can be the missing link in the development of MOF. Elevated levels of extracellular phospholipases, reactive oxygen and nitrogen species, pore-forming proteins (PFPs), and dysregulation of osmotic homeostasis occurring upon systemic inflammatory response are the major extracellular inducers of plasma membrane damage, which may simultaneously operate in different organs causing their profound dysfunction. Hypoxia activates similar processes, but they predominantly occur within the cells targeting intracellular membrane compartments and ultimately causing cell death. To combat the plasma membrane damage cells have developed several repair mechanisms, such as exocytosis, shedding, and protein-driven membrane remodeling. Analysis of knowledge on these mechanisms reveals that systemic damage to plasma membranes may be associated with potentially reversible MOF, which can be quickly recovered, if pathological stimuli are eliminated. Alternatively, it can be transformed in a non-resolving phase, if repair mechanisms are not sufficient to deal with a large damage or if the damage is extended to intracellular compartments essential for vital cellular functions.

## Introduction

Considerable number of critically ill patients develop multiple organ failure (MOF) (also called multiple organ dysfunction syndrome (MODS), which is the leading cause of morbidity and mortality in intensive care patients (1–3). Despite major advances in intensive care medicine, we still know very little about this syndrome. It is commonly accepted that MOF has a multifactorial character, based on two general pathological processes, namely, hypoxia and overwhelming inflammatory response (4, 5). Surprisingly, even with advanced organ dysfunction occurring in MOF, all the failed organs appear normal and manifest minimal signs of cell death (6, 7). Normal appearance of failed organs was observed even in patients who died of non-resolving MOF (4, 8, 9). Furthermore, it is striking that if critically ill patients recover from MOF, failing organs can recover relatively fast, even those organs that have poor regenerative capacity (10). There are some other unresolved questions about MOF highlighted elsewhere (9, 11, 12). The fact that the dysfunction occurs parallel in several organs suggests a similar pathologic mechanism(s) operating in quite different organs. The organ failure occurs despite the centralization of circulation, which increases blood flow in vital organs often occurring in critically ill patients. A decrease in oxygen consumption and ATP levels is observed in tissues, despite of adequate tissue oxygen supply. These unresolved questions suggest that the pathological changes causing MOF occur on molecular level and do not influence cellular morphology. Even electron microscopy did not reveal continuously reproducible data. In single studies, specific changes in the ultrastructure such as swollen mitochondria (13), delayed endoplasmic reticulum (ER) (6), and formation of autophagosomes (7) have been shown for specific, but not for all the cases and they were not reproducible in different studies/models. The manifestation of MOF described above is particularly characteristic for MOF mediated by severe inflammatory response, such as in septic patients. In contrast, hypoxia, accompanied by nearly complete inhibition of ATP synthesis, causes cell death and formation of necrotic areas in affected organs (14). The mechanism leading to necrosis upon hypoxia is much better understood than MOF mediated by systemic inflammatory response. According to our current knowledge, mechanisms causing MOF should be reversible at least at the beginning of the disease and they should induce cell dysfunction, but not cell death. They should undergo fast recovery pathway(s); the pathways of damage and recovery should operate in all the susceptible to failure organs and there should be a reasonable explanation, why increased blood flow through the organs (centralization of circulation) does not preserve organ function often even deteriorating the situation.

Strikingly, damage to plasma membranes impairs their barrier function as well as other important cellular functions. Impairment of these functions leads to serious and quickly developed cellular dysfunction (15, 16). Since integrity of plasma membranes is very important for all the cells, they have developed robust membrane repair mechanisms (15), which include exocytosis, endocytosis, ectosome shedding, and protein-driven membrane remodeling (15). The recovery of the phospholipid bilayer is the major aim of the cell membrane repair strategy. The damage to the plasma membrane impairs the entire cellular homeostasis, but if the mechanism of damage is terminated, then the membrane can quickly repair by the abovementioned efficient mechanisms (17). Over the last decade, the knowledge about the mechanisms of membrane damage and recovery has been strongly extended and describes in details a number of such mechanisms (18). It is striking that the majority of mechanisms damaging membranes obligatorily accompany MOF. Membrane disruptions can be caused by so-called pore-forming toxins (PFTs) [pore-forming proteins (PFPs)] secreted by most pathogenic bacteria (19). The pores formed by these proteins can be permeable not only for small molecules, but also for proteins (20). Hypoxia and reoxygenation can also induce membrane damage *via* changes in Ca^2+^ homeostasis and generation of intracellular reactive oxygen and nitrogen species (RONS), which cause widespread oxidation of both the proteins and lipids associated with membrane damage occurring predominantly in intracellular compartments (18, 21). It has been proposed that plasma neuronal membrane disruption is a major contributor to morbidity of neurological patients (22). The assumption that plasma membrane critically contributes to organ function satisfactorily explains MOF because mechanisms damaging biological membranes are universal in each organ. Below we consider the major mechanisms of membrane damage obligatorily accompanying MOF.

## Extracellular Reactive Oxygen and Nitrogen Species

**FIGURE 1:**
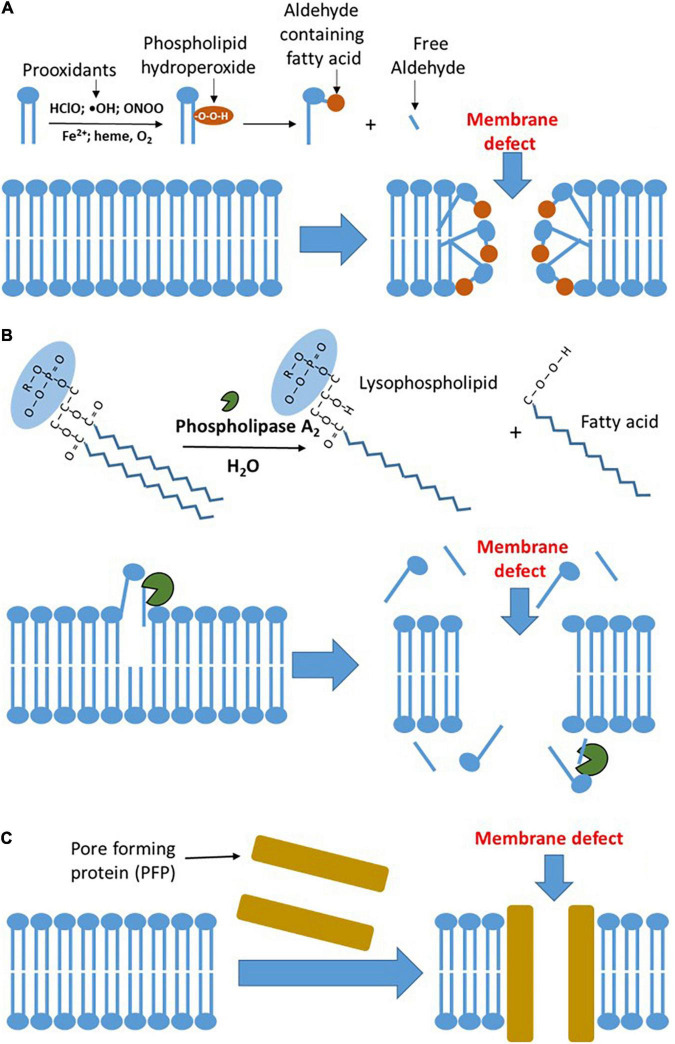
The major mechanisms of plasma membrane damage. **(A)** Oxidative damage to the plasma membrane. Prooxidants formed by inflammatory sources circulating in the blood induce lipid peroxidation in plasma membranes. The latter results in the formation of polar, hydrophilic species, which form defects in the membrane and increase its permeability. **(B)** Damage to the plasma membrane mediated by phospholipases. Elevated concentration of phospholipase A_2_ (PLA_2_) in the circulating blood hydrolyzes phospholipids in the plasma membranes. This reaction results in the formation of lysophospholipids and free fatty acids, which are prone to leave the membrane and increase its permeability facilitating lipid peroxidation in plasma membranes. The latter results in the formation of polar, hydrophilic species, which form defects in the membrane and increase its permeability. **(C)** Pore-forming proteins (PFPs) mediated damage to plasma membrane. Upon binding to lipid membranes, they convert from the soluble form into an oligomeric state, undergo conformational change, and form transmembrane pores, which dramatically increase the membrane permeability. HClO, hypochlorite; ONOO, peroxynitrite; and OH, hydroxyl radical.

Upon systemic inflammation, RONS formed by immune cells is released into extracellular fluid to attack pathogens, but also host cells in case of overwhelmed activation of immune cells. Superoxide radicals (O_2_^•–^) are the primary reactive species generated by immune cells. It can be converted into hydrogen peroxide (H_2_O_2_) by superoxide dismutase (SOD) or *via* spontaneous dismutation. Both the O_2_^•–^ and H_2_O_2_ are not very aggressive species and serve predominantly for signaling purposes (23). However, O_2_^•–^ and H_2_O_2_ contribute to the generation of two chemically aggressive species, peroxynitrite (ONOO) and hypochlorite (HClO), respectively. ONOO and HClO are used by immune cells to kill bacteria, but they can also damage host cells in the place they are produced (23). They cannot move to another location due to their high reactivity and short half-life time. In contrast, H_2_O_2_ can diffuse at longer distances from the place it was formed. H_2_O_2_ can induce oxidative stress upon reaction with heme iron or ferrous ions. This, so-called Fenton reaction, yields hydroxyl radical (HO^•^), which is a very reactive species activating lipid peroxidation and damaging biological membranes. Free ferrous ions occur only within the cells, while heme iron is predominantly present in the blood in a form of hemoglobin (24). In the blood, hemoglobin bound to haptoglobin (cell-free hemoglobin) and is the major catalyzer of the Fenton reaction (25). It has been shown that elevated levels of cell-free hemoglobin are associated with an increased risk of death in septic patients (26). This effect is attributed to the induction of oxidative stress, which impairs endothelial permeability, in turn having particularly dramatic consequence in the lung (26). Moreover, H_2_O_2_ can also undergo a redox reaction with methemoglobin yielding ferryl species, which are extremely reactive pro-oxidants and they are prone to damage host cells (27). The major target of RONS in plasma membranes is the lipid bilayer. RONS induce lipid peroxidation in biological membranes resulting in the formation of polar oxidation products, which increase the permeability of biological membranes (Figure 1A).

## Intracellular Reactive Oxygen and Nitrogen Species

Reactive oxygen and nitrogen species are released not only in extracellular fluids, but also within the cells. Generation of intracellular RONS is predominantly associated with the mitochondrial electron transport chain generating O_2_^•–^ and nitric oxide (NO) synthases (NOSs), a family of enzymes generating NO. Both the O_2_^•–^ and NO serve as intracellular messengers, if produced in physiological amounts (23, 28). The levels of mitochondrial RONS are elevated upon the systemic inflammatory response *via* a mechanism called RONS-NOS cycle (29), which comprises several steps. First, inflammatory mediators upregulate inducible NOS (iNOS) causing drastic increase in the intracellular NO levels. NO reversibly binds to complex IV at the same site as the oxygen (30). This inhibits the electron flow through the mitochondrial electron transport chain to complex IV and its two electron reduction to water (31). Instead, the electrons leak to oxygen from complexes I and III being reduced by one electron to O_2_^•–^ (29). The intracellular interaction between NO and O_2_^•–^ induces mRONS-NOS vicious cycle mentioned above, which continuously elevates cytoplasmic levels of O_2_^•–^ and NO until their levels are sufficient to form ONOO and damage cellular membranes causing the release of intracellular enzymes into extracellular fluid (29). This is accompanied by a drop in cellular ATP levels impairing the entire cellular metabolism. In specific cell types and under hypoxic conditions, xanthine oxireductase (32) and NADPH-oxidase also contribute to the intracellular RONS pool, predominantly by generation of H_2_O_2_ (33). NADPH oxidase also contributes to the crosstalk with mitochondria *via* RONS generated at both the sites (33, 34).

An additional mechanism activating oxidative stress upon hypoxia is the release of ferrous ions from the ferritin. Ferrous ions are extremely strong activators of oxidative stress, particularly of lipid peroxidation (LPO) (35). Iron-mediated LPO can irreversibly damage intracellular compartments such as mitochondria; this action of iron can be prevented by chelation of ferrous ions, for instance, by NO (36). Of note, NO can be deleterious in the presence of oxygen upon inflammation forming ONOO and beneficial under hypoxic conditions inactivating ferrous ions. There are clinical observation supporting the key role of RONS. It has been shown that common antioxidant therapies reduce MOF and inflammation (37). *In vivo*, it is difficult to dissect the effects of inflammation and hypoxia and they often appear together due to systemic interactions. Precisely cut liver slices, which maintain tissue structure, but are free of systemic influences, can be used to separate effects of inflammation and hypoxia in the tissue. In these slices, it has been shown that either treatment with a cocktail of inflammatory mediators or hypoxia induce the release of liver damage markers aspartate aminotransferase/alanine aminotransferase (AST/ALT), while mitochondrial dysfunction was observed only under hypoxia (38). This supports the assumption that inflammatory mediators predominantly impair plasma membrane, while hypoxia affects both the plasma membranes and intracellular compartments.

## Extra- and Intracellular Phospholipases

Phospholipases are a family of enzymes that cleave ester bonds within phospholipids. This hydrolytic reaction releases free fatty acids and lysophospholipids. These products control a number of cellular signaling pathways. Phospholipase A (PLA) occurring in all the human cells is particularly often involved in pathophysiological processes (39). PLA is a family of enzymes including several forms. The Ca^2+^-dependent cytosolic and secreted PLA_2_ as well as Ca^2+^-independent PLA_2_ are predominantly associated with physiological and pathological processes (40, 41). PLA_2_ is also the major toxic component of snake venom (42). Extracellular (secreted) PLA_2_ belongs to the acute phase proteins and releases in response to inflammatory stimuli. PLA_2_ hydrolyzes glycerophospholipids in position two and destabilizes lipid part of the membranes increasing membrane permeability. This reaction, called phospholipolysis, is essential for bactericidal activity (43). The enzyme type IIA of the secreted phospholipase A2 (s PLA_2_-IIA) is particularly important for the mammalian innate host defense against bacterial infection (44). The expression of PLA_2_ in mammalian cells is regulated by proinflammatory cytokines, such as interleukin-1 (IL-1) and tumor necrosis factor-alpha (TNF-alpha) (45) *via* nuclear factor-kappa B (NF-κB) and PPARg pathways (46). PLA_2_ is not specific for bacterial phospholipids and hydrolyzes phospholipids in all the membranes, including host cell membranes.

The levels of PLA_2_ are drastically increased in septic patients (47) and the high levels of PLA_2_ are associated with the increased mortality in these patients (48). This supports the assumption that systemic damage to plasma membranes is critical for outcome of septic patient. PLA_2_ also plays a pivotal role in the development of acute respiratory distress syndrome (ARDS) (49). Activation of PLA_2_ upon inflammation is very fast; for instance, IL-1 activates intracellular PLA_2_ within several minutes (50). Activation of intracellular PLA_2_ damages mitochondrial membranes (51). Mitochondrial dysfunction results in a decrease in ATP levels, subsequently increases cytosolic calcium levels, which further activates phospholipase. Accumulation of lipid breakdown products, unesterified free fatty acids, acyl carnitine, and lysophospholipids, which have a detergent effect, further aggravate membrane damage and induce the leak of intracellular content into extracellular fluids (52). Thus, the spectrum of phospholipase actions is ranging from contribution to regulatory pathways and defense against bacterial infection to the damage of host cell membranes and subcellular organelles. It has been shown that septic mice, pretreated with GW4869, an inhibitor of PLA_2_, ameliorate the disease decreasing the levels of circulating cytokines and extracellular vesicles elevated in response to sepsis (53). An overview of PLA2-mediated membrane damage is given in Figure 1B.

## Direct Damage by Host and Pathogen Proteins

The third mechanism of membrane damage accompanying the development of MOF is the interaction of membranes with so-called PFTs, also termed as PFPs. These proteins disturb biological membranes and increase permeability by forming large pores (18) (Figure 1C) due to cytolytic transmembrane assemblies; this not necessarily kills the cells, but substantially changes cellular functions (54). PFPs induce the influx of calcium and efflux of potassium, altering intracellular ion homeostasis. Particularly critical are elevated Ca^2+^ levels because PFPs strongly facilitate the influx of Ca^2+^ and its levels quickly become toxic (55, 56) causing desensitization of immune cells (57), destabilization of tissue barriers, and subsequently leading to organ failure (58). PFPs are best characterized in bacteria (18) and they are the largest group of bacterial toxins comprising about 30% of all the bacterial toxins (59). PFPs have been shown to play a critical role in the initial stages of neonatal sepsis and to be associated with poor clinical outcome (60). PFPs also occur in eukaryotic cells. The mammalian immune system has adopted PFPs to kill pathogens (61). PFP gasdermin D is active in neutrophils and plays a crucial role in the release of neutrophil extracellular traps (62). However, excessive formation of these traps aggravates organ failure in septic patients, while its inhibition attenuates MOF (62). Another important PFPs formed by the mammalian immune system are the complement components (C5, C6, C7, and C8), also named as membrane attack complex (MAC) (63). Excessive activation of the complement system has also been attributed to the worsening of MOF (64, 65).

## Osmotic Shock and Mechanical Damage

Osmotic dysregulation causes severe cell damage resulting in cell swelling and ultimately cell lysis and necrotic cell death. Osmotic damage occurs due to disturbance of the electrolyte balance, particularly due to pathologic changes in potassium and sodium homeostasis as well as in extracellular fluid volumes. Water/electrolyte balance disorders are commonly found in hospitalized patients, most often in the elderly patients (66) and are associated with an increased risk of death (67). The kidneys play a particularly important role in the regulation of body water and osmotic balance and they are very susceptible to failure in patients presenting hypernatremia (68). Hyponatremia has also been associated with an increased mortality following bacterial infectious diseases (69). It has been shown that both the hypernatremia (70) and hyponatremia (71) increase the risk of death. Severe hyponatremia is directly associated with the development of MOF (72).

In addition to osmotic, also mechanical deformation of tissues may damage membranes. Mechanical deformation may appear due to external blast or lateral tension appearing upon trauma or excessive physical exercises. Muscle fibers are particularly prone to injury mediated by mechanical deformation (73). Muscle damage can also appear in patients suffering from muscular dystrophy, due to changes in cytoskeletal protein, dystrophin (74). Osmotic shock and mechanical deformation may aggravate organ dysfunction induced by hypoxia and inflammation. For instance, it has been shown that in patients with mechanical trauma, the overwhelmed activation of complement is the predictor of MOF (75).

Thus, there are numerous mechanisms potentially causing membrane damage, which are associated with MOF. The mechanisms connecting damage of plasma and intracellular membranes and organ function/dysfunction will be considered below.

## Consequences of Damage to Plasma Membranes

Plasma membrane integrity is obligatory for physiological cellular homeostasis; the impairment of the plasma membrane causes dysregulation of ion homeostasis and the release of cellular content in extracellular fluids (76). Moderate damage to the plasma membrane does not lead to cell death, but affects cellular functions and communication between the cells (76–78), both are important for organ function and they are almost reversible. These reversible changes often occur in muscle tissues, which are particularly susceptible to damage during exercises and the body has well-developed mechanisms repairing this damage. However, in severe local trauma, such as traumatic brain injury, trauma exceeds the repair capacity of the cells and this results in membrane lesions and severe neuronal dysfunction (79). The damage to cell membranes caused by microbial pathogens and immune cells can also exceed repair capacity and can have deleterious consequences for cellular functions (58, 80).

It is still unclear how to determine critical size of the membrane damage and compare it with potential repair capacity as well as how different mechanisms of membrane repair cooperate in order to recover membrane integrity (76). The progress in this field is limited by a number of technical restrictions. The major technical problem is that visualizing and characterizing damages to plasma membrane are difficult due to low resolution of analytical methods and rapid speed of damage and repair processes (76). In the majority of the studies, the damage to plasma membrane is determined by three indirect means, including the entry of cell-impermeable molecules, calcium influx, and the release of intracellular contents into the extracellular fluid (81). However, already these approaches have delivered a number of examples showing that instability of plasma membranes is a prerequisite of MOF. Intestinal epithelial injury determined by entry in the tissue of 4 kDa fluorescein-dextran has been shown to predict subsequent development of MOF (82). Hyperpermeability of endothelium triggered by inflammation or ischemia promotes edema, exacerbating disease progression, and slowing down recovery (83). Increased permeability of endothelium allows the inflammatory mediators/acute phase proteins diffuse into tissue (84) extending the process of disintegration of plasma membrane to parenchymal cells in different organs. Increased permeability of plasma membranes for Ca^2+^ is particularly deleterious for severe patients. This is supported by the fact that infusion of Ca^2+^ exacerbates organ failure and mortality in septic patients (85). The leak of potassium from cells into extracellular fluids also has a critical pathological impact. Hyperkalemia is associated with poor outcomes of patients in different pathological settings, including the acutely ill patients (86); the primary mortality risk in these patients is cardiac electrophysiological disturbances (87).

**FIGURE 2:**
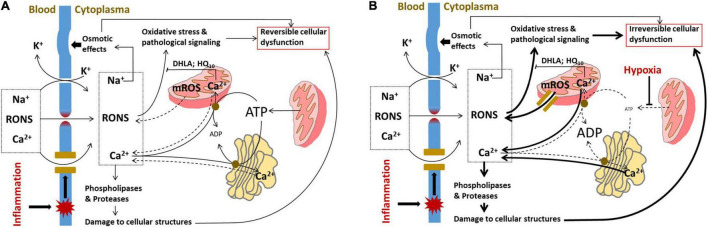
Induction of cellular dysfunction occurring due to damage to cellular membranes. **(A)** Pathologic mechanisms operating in parenchymal cells upon damage to plasma membrane. The influx of RONS and Ca^2+^ through the defects in plasma membrane is partially compensated by intracellular organelles, which scavenge RONS and take up Ca^2+^ from the cytoplasm by ATP-dependent Ca^2+^ pumps. In addition, the influx of Na^+^ and leak of K^+^ are compensated by Na^+^/K^+^ pump, which uses ATP to pump the ions (not shown). At this phase of disease, the cells are exposed to oxidative stress, osmotic shock, and elevated activities of phospholipases and proteases; however, all these changes are still reversible because if sources of damaging substances in the blood are removed, the plasma membrane will be repaired and the functional intracellular organelles will restore the normal cellular metabolism. **(B)** Pathologic mechanisms operating in parenchymal cells upon damage to plasma membrane combined with damage to intracellular compartments induced by hypoxia. Under hypoxic conditions, the ATP levels in cells are drastically decreased and all the ATP-dependent processes are slowed down. The mitochondria instead of taking up Ca^2+^ and scavenging RONS will release Ca^2+^ and RONS; the latter leak through permeability transition pore, an intracellular PFP formed in mitochondria. The pathological changes in ion homeostasis are decompensated and Na^+^/K^+^ imbalance will be further aggravated. The cell comes in a kind of decompensation phase and the cellular dysfunction will become irreversible. RONS, reactive oxygen and nitrogen species; DHLA, dihydrolipoic acid; HQ_10_, ubiquinone; and mROS, mitochondrial reactive oxygen species.

The damage to the plasma membrane and influx of extracellular Ca^2+^ can be at least partially compensated by intracellular organelles. RONS can be scavenged by intracellular antioxidant systems and excess of Ca^2+^ can be removed from the cytoplasma by ATP-dependent Ca^2+^ pumps in the ER and the mitochondria (Figure 2A). After repair of the plasma membrane, cell functions can be recovered.

## Consequences of Damage to Intracellular Membranes

The integrity of intracellular compartments is critical for vital cellular functions. Impairment of both the outer and inner mitochondrial membranes compromises mitochondrial function. Damage to outer mitochondrial membrane causes the release of cytochrome c. This inhibits electron transport along mitochondrial electron transport chain to oxygen and consequently inhibits ATP synthesis. Released cytochrome c can also induce apoptosis. Increased permeability of the inner mitochondrial membrane does not affect the electron transport, but reduces mitochondrial membrane potential and uncouples oxidation from phosphorylation causing a drop in ATP synthesis. The formation of the mitochondrial permeability transition pore, a kind of intracellular PFP, is considered as the major mechanism of uncoupling (88). Iron-mediated lipid peroxidation is another mechanism impairing outer mitochondrial membrane leading to the release of cytochrome c under hypoxic conditions (36). Mitochondrial dysfunction has been suggested to cause MOF upon systemic inflammation (89, 90), although it may be the result of secondary hypoxia (38). Irrespective of the underlying mechanism, patients with sepsis have better prognosis, if they have higher levels of tissue ATP (91). Decreased levels of ATP cause the release of Ca^2+^ and activation of numerous intracellular phospholipases and proteases, which normally exert only a modest activity.

However, low levels of ATP can also be beneficial by protecting against apoptosis. Oxidative damage to ER often induces so-called ER stress. ER stress during critical illness has been less thoroughly studied than mitochondrial dysfunction, but it has been shown to play a critical role in a number of acute pathologies accompanied by MOF (92). In animal models, markers of increased ER stress have indeed been observed in heart and liver during sepsis (92) as well as in failing organs following hemorrhage, trauma, and ischemic injury (92–95). In animal models accompanied by MOF, ER stress markers were associated with organ failure (96, 97) suggesting that ER stress contributes to the development of MOF. The effect of ER stress on function of different organs during critical care diseases needs to be further investigated. A critical situation appears, if the plasma membrane damage occurs simultaneously with hypoxia. Hypoxia slows down the mechanisms compensating damage to the plasma membrane. Mitochondrial permeability transition pore formed under hypoxic conditions facilitates the release of mitochondrial ROS and cytochrome c into cytoplasm. The decrease in ATP synthesis results in the release of Ca^2+^ from intracellular storage (mitochondria and ER) instead of taking Ca^2+^ up from cytoplasm. We assume that overwhelmed activation of intracellular Ca^2+^-dependent phospholipases and proteases along with excessive RONS generation are the major mechanisms causing irreversible cell damage (Figure 2B).

## Mechanisms of Membrane Repair

**FIGURE 3:**
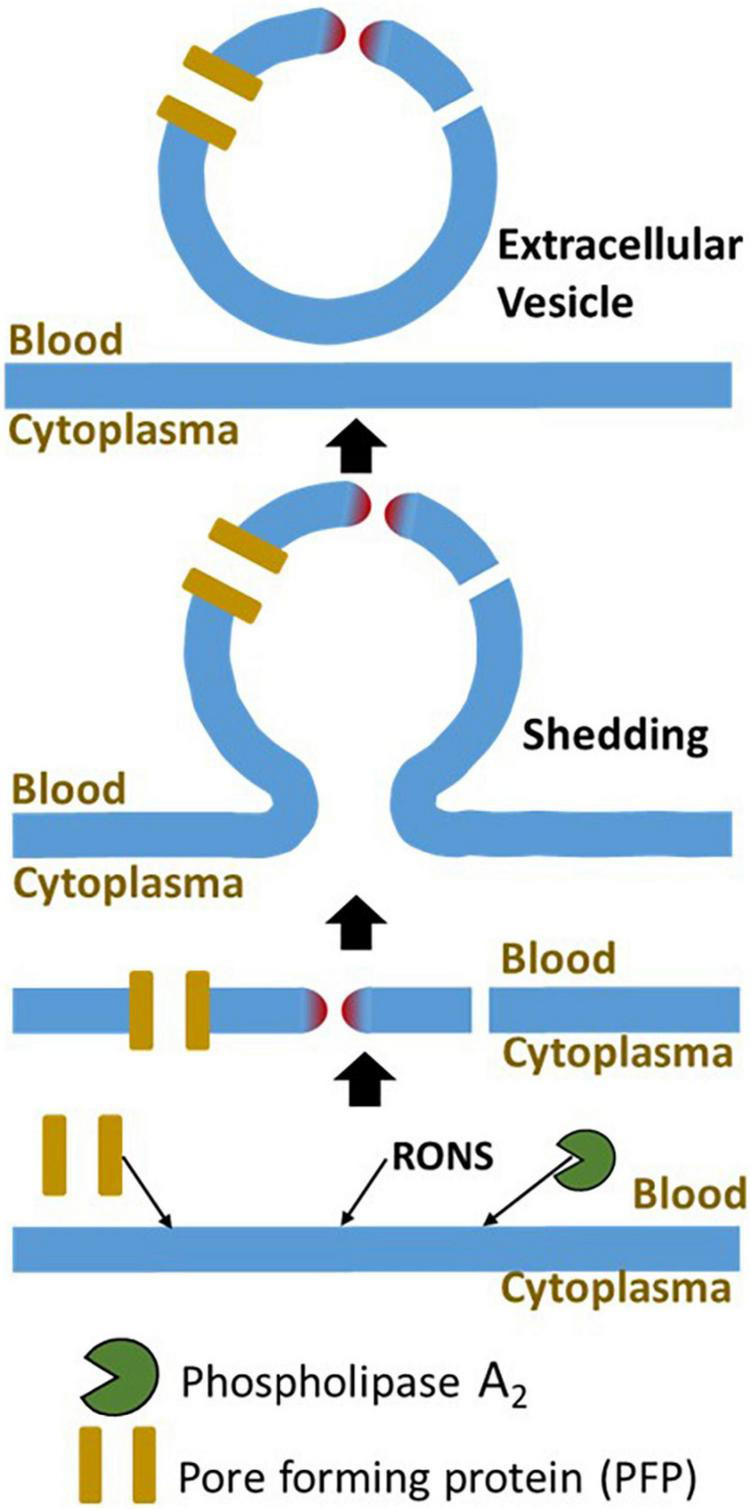
The major mechanisms of damage to plasma membrane and its repair. The plasma membrane is damaged by three major mechanisms, interaction with PFP, lipid peroxidation, and elevated phospholipase activity. The major mechanism repairing of this damage is membrane shedding. The damaged part of the plasma membrane will be excluded from the cell in form of an extracellular vesicle. RONS, reactive oxygen and nitrogen species.

Membrane defects can be efficiently repaired, if they do not exceed the critical size of the damage. Membrane fusion and replacement are two main strategies to repair the plasma membranes (15, 18). Membrane fusion is realized by exocytosis, while the removal of damaged membranes is executed by endocytosis or shedding. The closure of membrane defect can be achieved *via* protein-driven membrane remodeling and wound closure (16, 98, 99). Repair mechanisms can be activated in a very fast mode; in some cases, exocytosis can be activated for less than 1 min (100). The activation of exocytosis and shedding results in an increase in the quantity of so-called extravascular vesicles (EVs) into blood or other extracellular fluids (Figure 3). EVs consist of exosomes (∼40–200 nm), microvesicles (∼200–1,000 nm), and apoptotic bodies (500–3,000 nm), a nomenclature based on the biogenesis pathways (101).

Judging from patterns of EVs, one can distinguish between activation of repair (shift to small EVs) or death pathways (shift to large EVs). It has been shown that EV sizes in septic patients and cecal ligation and puncture (CLP) mice are remarkably smaller than in corresponding controls (102, 103), suggesting that in septic patients, the membrane repair process rather than apoptosis is initiated as a response to plasma membrane damage. Indeed, except lymphocytes, no other apoptotic cells have been reported in sepsis patients (7) and animals (6) even in those dying from sepsis. The role of EVs in sepsis is controversial; it has been associated with both the proinflammatory (102, 104) and anti-inflammatory (105) effects. The literature on metabolic effects of EVs in sepsis was recently reviewed in detail by Burgelman et al. (101). Intriguingly, in septic patients, significantly higher levels of endothelial-derived EVs were determined in the blood of survivors compared to non-survivors (106). This is in line with the assumption that endothelium membranes are the primary targets for membrane damage upon systemic inflammatory response and consequently repair mechanisms are first activated in those cells.

## Regulation of Membrane Repair

There are several mechanisms regulating cell repair. As mentioned above, inflammatory response is accompanied by the release of PLA_2_, which on one hand damages biomembranes, but on the other hand activates exocytosis, a repair mechanism. The critical role in the repair activation plays soluble *N*-ethylmaleimide-sensitive factor attachment protein receptor (SNARE) proteins. SNARE is responsible for the connection between vesicular and plasma membranes; this connection is essential for activation of exocytosis facilitating the fusion between vesicles and the plasma membrane (107). The fusion with the damaged plasma membrane is facilitated by polyunsaturated fatty acids (PUFAs) generated from PLA_2_ activity. Thus, PLA_2_ activity has a double function. On one hand, it damages the membranes, but simultaneously it can induce repair processes in a product-dependent manner. For instance, it has been shown that PUFAs have a protective effect recovering increased permeability of endothelium. Arachidonic acid and sphingosine are particularly strong enhancers of exocytosis (108, 109). More details about interaction between PUFAs and SNARE can be found in the review by Virginia Garcia-Martinez (110). The second class of molecules regulating membrane repair are RONS. Similarly to PLA_2_, RONS are involved in both the membrane damage and regulation of membrane repair. RONS facilitate exocytosis by specific activation of lysosomal TRPML1 (the first member of the mammalian mucolipin TRP channel subfamily) channels, inducing lysosomal Ca^2+^ release, which, in turn, stimulates lysosomal exocytosis *via* calcineurin, a Ca^2+^-dependent phosphatase (111). Activation of repair mechanisms by RONS depends on their concentration. At low concentrations, RONS act as a second messenger stimulating lysosomal exocytosis and facilitating membrane repair. In contrast, high levels of RONS inhibit lysosomal exocytosis manifesting exclusively damaging potential (112). The process of repair is also facilitated by Ca^2+^ influx through injured plasma membrane areas. Influxed Ca^2+^ has two functions. First, it facilitates the fusion of preexisting intracellular vesicles to form so-called membrane “patches.” Second, the location of Ca^2+^ influx indicates the place of the membrane damage and facilitates the fusion of membrane “patches” with the injured part of the plasma membrane using Ca^2+^ as an anchor (113).

## Discussion

**FIGURE 4:**
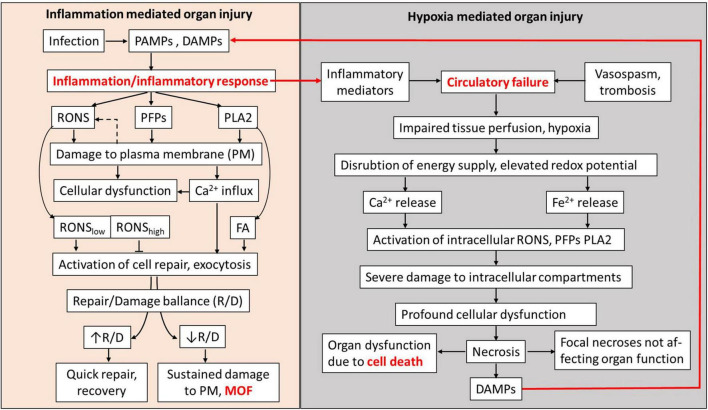
Schematic presentation of MOF pathogenesis mediated by plasma membrane damage. RONS, PFPs, and PLA_2_ released in blood and extracellular fluids upon inflammatory response cause damage to plasma membranes (PMs) of well-perfused organs. Influx of Ca^2+^, RONS, and products of PLA_2_ simultaneously activate PM repair mechanisms. RONS may also be released from cells (dashed arrow). Damage and the counteracting repair establish a repair/damage balance, which is critical to define whether involved organs will recover or undergo MOF. Hypoxia occurring concomitantly with inflammatory response causes the increase in RONS and Ca^2+^ levels and PLA_2_ activity inside the affected cells, leading to severe damage of intracellular organelles causing cell death and the release of DAMPs. Inflammatory response and hypoxia interact *via* the release of inflammatory mediators and DAMPs. In well-perfused organs, the damage to PM and development of MOF have higher impact than hypoxia-mediated damage to intracellular compartments. PAMPs, pathogen-associated membrane patterns; DAMPs, damage-associated molecular patterns; RONS, reactive oxygen and nitrogen species; PFPs, pore forming proteins called also protein forming toxins (PFPs); PLA_2_, phospholipase A_2_; FA, fatty acids; R/D balance, repair/damage balance; PM, plasma membrane; and MOF, multiple organ failure called also multiple organ dysfunction syndrome (MODS).

In this review, we have considered two general pathological processes, systemic inflammatory response and hypoxia, which are associated with MOF and collected evidence that the release of substances causing damage to plasma membranes in diverse organs is a key process leading to MOF (Figure 4). Inflammatory response causes damage to plasma membranes mediated by elevated levels of PLA_2_, RONS, and PFPs in extracellular fluids. The damage to plasma membranes is potentially reversible and the rate of recovery depends on the size of the damage and the repair capacity. Similar mechanisms mediated by PLA_2_, RONS, and PFPs are induced by hypoxia. However, upon hypoxia, these mechanisms are activated within the cells and their primary targets are intracellular compartments, such as mitochondria and ER, which are essential for vital cellular functions. Damage to these organelles causes irreversible cellular dysfunction and ultimately leads to cell death. We assume that the focal necrosis sometimes observed in severe preclinical models of systemic inflammatory response or sepsis is due to secondary hypoxia rather than to the action of inflammatory mediators. Since repair of plasma membranes is a fast process, it can be achieved in a short time after elimination of pathologic stimuli.

Membranes have similar structure in all the organs; that is why, several organs can be affected simultaneously. The circulatory centralization often occurring upon shock ameliorates oxygen supply to vital organs, but simultaneously delivers more inducers of plasma membrane damage (RONS, PLA_2_, and PFPs); that is why, damage appears despite improved tissue perfusion. It looks like the body decides to induce reversible dysfunction caused by inflammation in several organs to avoid the “death” of single organs induced by hypoxia. This can be a good solution for short-term acute critical phases, but on the long run, this causes the death of the whole body due to systemic dysregulation induced by MOF, although each failed organ would have a chance to recover.

Although MOF is not characterized by noticeable death of parenchymal cells in failed organs, it is accompanied by elevated death of immune cells. The programmed death of immune cells was associated for a long time with apoptosis induced by the damage to mitochondrial membranes and the release of cytochrome c (114). More recently, the death of immune cells was attributed to necroptosis, which is activated by the inflammatory mediator, TNF-alpha. This pathway has already been attributed to critical care diseases and it is characterized by the loss of plasma membrane integrity (115); consequently, this supports the key role of plasma membrane damage in the development of MOF. As we mentioned above, the hypoxic conditions facilitate the release of ferrous ions from the ferritin and can potentially activate ferroptosis, a programmed cell death regulated by iron-containing compounds (116). Indeed, recently ferroptosis was associated with the vascular leakage upon septic conditions (117). Damage to intracellular compartments such as mitochondria stimulates autophagy and formation of autophagosomes. It has been shown that autophagy is activated in septic patient and animal models of sepsis (7). Moreover, autophagy has been shown to play a protective role against sepsis (118). We assume that the release of damaged membranes with exosomes formed in autophagosomes is the mechanism underlying beneficial effects of autophagy.

We are convinced that current knowledge on membrane damage upon MOF summarized here will attract attention of researchers and clinicians to this field stimulating basic and clinical research and creating the basis for novel powerful diagnostic and therapeutic tools improving clinical outcome of patients with MOF.

## Author Contributions

AK conceived the review, drafted the manuscript and the figure, and approved the final version of the manuscript. JG wrote the section on extravascular vesicles and approved the final version of the manuscript. Both authors contributed to the article and approved the submitted version.

## Conflict of Interest

The authors declare that the research was conducted in the absence of any commercial or financial relationships that could be construed as a potential conflict of interest.

## Publisher’s Note

All claims expressed in this article are solely those of the authors and do not necessarily represent those of their affiliated organizations, or those of the publisher, the editors and the reviewers. Any product that may be evaluated in this article, or claim that may be made by its manufacturer, is not guaranteed or endorsed by the publisher.
